# Development of Virtual Reality Systems for Social Skills Training in Individuals with Autism Spectrum Disorder: A Systematic Literature Review

**DOI:** 10.1192/j.eurpsy.2025.1436

**Published:** 2025-08-26

**Authors:** H. Masullo, M. Galloro

**Affiliations:** 1 Unisa, São Paulo, Brazil

## Abstract

**Introduction:**

Autism Spectrum Disorder is a neurodevelopmental condition characterized by deficits in social communication, restricted and repetitive patterns of behavior. In recent years, there has been a growing interest in using virtual reality technologies for social skills training in individuals with ASD. Virtual reality offers the opportunity to create immersive and interactive virtual environments where participants can practice and enhance their social skills in a safe and controlled setting.

**Objectives:**

To analyze the quality of using virtual reality as a treatment method for patients with autism spectrum disorder to improve their social skills.

**Methods:**

Through a descriptive systematic review, studies in Spanish and English from the years 2021 to 2024 were analyzed, selected from the databases PubMed, Scielo, and Lilacs, using the keywords: “Virtual reality”; “Autism spectrum disorder”; “Social skills”. Articles that did not address virtual reality as the main training method, were off-topic, or did not present results from this method were excluded.

**Results:**

**Children and Social Skills**

Programs like FaceMe use gamified environments to teach facial expressions, while tools like GameBook use interactive scenarios and augmented reality to reinforce these skills. VR’s ability to track and analyze eye movement patterns offers insights into social behavior. Programs like “Virtual Farm” and VR games such as Zentastic have demonstrated positive effects, with children engaging in various virtual social scenarios. Programs like VRESS use immersive scenarios to teach social skills that provide real-time feedback on behavior can help individuals develop self-awareness and adjust their interactions.

**Adults and Social Skills**

Adults with autism face challenges in non-verbal communication, understanding social cues, and forming relationships, which can lead to social isolation. VR offers simulations for practicing social interactions, enhancing communication, empathy, and conflict resolution. Programs like VRESS use immersive scenarios to teach social skills, and tools that provide real-time feedback on behavior can help individuals develop self-awareness and adjust their interactions.

**Conclusions:**

The development of virtual reality systems for social skills training in individuals with autism spectrum disorders represents an innovative and promising approach to assisting in the development of these skills in both children and adults with autism. Virtual reality offers a more engaging and effective training experience, allowing users to practice social skills in a controlled environment tailored to their individual needs. Despite the promising results, there are still challenges to be faced, such as methodological issues and the need for awareness and training of health and education professionals.

**Disclosure of Interest:**

None Declared

